# Sustainability of Household Food Waste Reduction: A Fresh Insight on Youth’s Emotional and Cognitive Behaviors

**DOI:** 10.3390/ijerph18137013

**Published:** 2021-06-30

**Authors:** Saman Attiq, Ka Yin Chau, Shahid Bashir, Muhammad Danish Habib, Rauf I. Azam, Wing-Keung Wong

**Affiliations:** 1 Air University School of Management, Air University Islamabad, Islamabad 54000, Pakistan; saman.attiq@mail.au.edu.pk; 2 Faculty of Business, City University of Macau, Macau 999078, China; gavinchau@cityu.mo; 3 Business Studies Department, Namal Institute, Mianwali 42250, Pakistan; shahid.bashir@namal.edu.pk; 4 Department of Business Administration, Air University Islamabad, Aerospace and Aviation Campus Kamra, Attock 43350, Pakistan; danish.habib@aack.au.edu.pk; 5 Punjab University of Technology Rasul, Mandi Bahauddin 50380, Pakistan; rauf.i.azam@gmail.com; 6 Department of Finance, Fintech & Blockchain Research Center, and Big Data Research Center, Asia University, Taichung City 41354, Taiwan; 7 Department of Medical Research, China Medical University, Taichung City 40447, Taiwan; 8 Department of Economics and Finance, The Hang Seng University of Hong Kong, Hang Shin Link, Siu Lek Yuen 41354, Hong Kong, China

**Keywords:** waste management, sustainable food waste reduction, household behaviors, anticipated guilt, perceived busyness, developing countries

## Abstract

The sustainability of food waste is one of the most important contemporary economic, social, and environmental issues that encompasses useful academic, practical, and policymaking implications. Under the domain of sustainability, food waste is a serious global challenge with a growing public, political, and corporate concern. Existing literature regarding the sensitization of consumers and the promotion of waste cautious behaviors still has much room for improvement in household waste. To bridge the gap in the literature, this study identifies and examines determinants of young consumers’ food waste reduction behavior in households. Using a sample size of 391 young consumers of household food products from Pakistan, a full-scaled administrative survey is conducted, and our hypotheses are empirically tested by using the PLS structural modeling equation. Our findings reveal significant impacts from both cognitive and emotional aspects on sustainable food waste reduction behavior. Our results have several important implications for policymakers and all the stakeholders, especially for marketers, including advertising strategies, policies to mitigate the impact of food waste, and the development of educational programs related to food waste.

## 1. Introduction

It is important to address the issue of sustainability because sustainability raises the question of whether future generations can have the same or larger resource basket as the current generation. Our resources could be consumed more by this generation and less would be left to the future, and this would be unreasonable [1]. The way products are produced and consumed has a great impact on the planet’s resources [2]. The over-consumption of natural resources by the present generation is an injustice which leaves future generations empty-handed. In the realm of marketing, marketers and companies have operated on the assumption of infinite resources with no regard to the harm caused to resources and the environment [3]. It has often been realized later that such activities are detrimental for many social, environmental, and economic factors. These activities also have the potential to cause irretrievable damage to the life of future generations and the environment [4]. Many people are worried that the environment will be irreparably damaged and the quality of life for future generations will be compromised as a result [5]. Additionally, established scientific traditions based on reductionist cause–effect relationships are incapable of explaining and addressing the complex dynamic interrelationships between economic, environmental, social, and temporal dimensions [6]. The ongoing damage to the environment and natural resources has necessitated a rise in demand for change in the manner of how we go about producing, marketing, as well as using products [7,8]. The growing public, political, and corporate perspectives on food waste are significant worldwide issues [9,10,11]. Not only is food waste an environmental issue for developing and developed nations, but is also a social and political challenge. Recent estimates have shown that world food waste per year stands at USD 1 trillion, causing not only economic burdens but also food insecurity [12]. It was estimated that food wasted in developing countries is worth USD 310 billion annually, which wastes large quantities of resources such as capital and energy, and puts constant stress on natural resources such as water and land [13]. These economically avoidable food losses have a negative impact on the revenues of both parties in the food supply chains and have also led to monetary losses for individuals and national economies [14]. Food waste has a significant impact on people’s food security from a social standpoint [1,15]. It was found that, globally, more than 800 million people were suffering from hunger or facing chronic undernourishment [16]. Food security issues can be addressed by reducing food waste sustainability and balanced nutrition food [17]. The availability and access to healthy food are the basic necessities for the well-being of individuals, society, and nations [18]. Other than these, food waste has an adverse impact on the environment. The prevalence of limited resources (e.g., workforce, soil, water) and the excessive use in food production of fertilizer and pesticides damage the natural environment [16]. According to Thyberg and Tonjes [18], food waste in landfills converts to greenhouse gases such as methane, having 25 times greater global warming potential as compared to carbon dioxide on a 100-year time scale. Food waste with serious threats for economic, social, and environmental aspects requires a sound understanding of food consumption and wastage patterns for the development of organized systems for waste management [2,19,20]. 

### 1.1. Role of Households in Food Waste

Food Waste is a major contributor to a serious threat for both humans and the environment; which requires well-organized systems for waste management [2,19,20]. Food security issues can be addressed by reducing food waste sustainability and balanced nutrition food [17]. It was found that approximately 1.3 billion tons of food (1/3 of which is suitable for human consumption) is wasted [21]. Research shows that around 36 million tons, accounting for 30% of food, is wasted in Pakistan [22]. Food waste studies revealed that households represent a significant amount of total food waste [23,24]. The end consumers are therefore one of the biggest contributors to the highest share of total food waste [25], particularly at the consumption stage [26]. The responsibility for the highest proportion of the total food waste [27] can be attributed to consumers and their consumption patterns. According to other research studies [27,28,29], people are aware of the issues with sustainable food waste, but unconscious practices lead to excessive food waste). Food waste in residences ranges from 28.4% to 31.9% [30], which appears to be higher than in other businesses. Consumers, regardless of being the major contributor of food waste [31,32], require a complex set of management behaviors [14]. Adding to the complexity, the existing research on sustainable food waste behavior in the context of household consumers is fragmented and provides contradictory findings, and thus, concluding these findings is very challenging [16]. However, encouragingly, some recent studies suggest that households can play a greater role in sustainable food waste management [33].

### 1.2. Young Consumers and Food Waste

It has been suggested that the younger generation requires careful monitoring to understand their behavioral intentions toward food waste in order to develop education programs to limit their food wastage practices [16]. Wakefield and Axon [34] highlighted the importance of behavioral intentions of unsustainable food practices among young consumers. The presence of a young age group in a household increases the volume of household food waste [35,36]. Young consumers are more concerned about the economic aspects than the health and environmental concerns of food waste [37]. Furthermore, the young consumer may not optimize their food consumption as they are not familiar with the reusing of leftover food [38].

Additionally, young consumers have little experience, and tend to miscalculate meal portion sizes, resulting in thrown away leftovers [39]. Some studies have explored the generational differences in food waste practices, and have found that millennials are a major contributor to food wastage and do not recognize the value of food [9,40,41]. [9,42] have also identified that young consumers waste more food compared to older ones. Conversely, [43,44] have argued that young consumers are more concerned about the wastage of food. Young people, especially students, are among the most profligate consumers of food in developed countries [2,3]). Young consumers were deemed appropriate as a subject of one study examining food waste behavior intentions [45,46], specifically in youth-dominant countries such as Pakistan. 

### 1.3. Research Gaps

Existing literature regarding consumer behavior with regards to household food waste opens several theoretical, methodological, and contextual gaps. Academic observers call for further research of food waste at a household–consumer level to address these existing gaps [10,47]. Researchers are increasingly focusing on unearthing the determinants of youth’s behaviors regarding sustainable food waste management as well as cost efficiency [48].

#### 1.3.1. Theoretical Gap

Theoretically, the complex nature of human behavior makes it very difficult to predict [49]. It has also been noted that behavioral studies mainly focus on cognitive variables such as informational appeals, normative aspects, perceived busyness, perceived behavioral control, awareness about consequences, and consumer perceptions towards sustainable food waste intentions [14,50]. However, in recent studies, it is suggested that emotional aspects are also involved during food consumption and its waste [51]. This research found that sustainable food waste may induce negative emotional aspects such as anticipated guilt and that individuals who felt guilty about food wastage are more active in reducing food waste sustainably [52,53]. Thus, consumers’ intentions toward food waste should be predicted using emotional factors such as anticipated guilt. It was also revealed that treating food waste as a social problem rather than just an economic and environmental issue improves the intensity of results [54]. Furthermore, researchers have suggested that external constraints are assumed to influence behavioral intentions and include interpersonal as well as structural factors such as limitations of time, resources, and infrastructure [55]. The value–action gap is the result of external factors such as social, political, and economic pressures [56]. Lack of time or perceived busyness is assumed as a barrier that may offset food waste reduction and limit the consideration to perform such behavior [57].

The existing body of knowledge extensively focuses on recycling and reuse behavior, whereas reducing waste behaviors are relatively ignored [58]. However, these behaviors offer financial [59], social [60], and potential environmental [61] benefits. Stancu, Haugaard [41] describes that if a person wishes to reduce his food expenditures, he must carefully acquire the food he requires and prepare and consume it as efficiently as possible. This may require relatively less effort than reuse and recycling. While, surprisingly, it was found that “reduction” is a key factor while reuse and recycling should be treated as secondary factors in waste-reducing strategies [59]. The inclusion of reduction is one of the necessary elements for waste prevention behavior, as reuse or recycling are not necessarily waste minimizers. To develop a more accurate picture of sustainable food waste behavior, the widely accepted concept of 3R (reduce, reuse, recycle) is preferred to the more traditional method of food waste intention [49,59].

#### 1.3.2. Methodological Gap

Methodologically, food waste reduction is recognized as one of the promising avenues in addressing the question of food waste [62]. It has been seen that food waste occurs at all stages in the supply chain, for example in agriculture, from production to distribution and via end-user [63]. An increasing number of works in the literature examine the behavior of waste from a consumer perspective and its factors [15,64]. In terms of consumers’ behavior towards food waste, the existing literature is less common than the research of food waste itself and the global impact of food systems [9]. [52] claimed that research on waste behavior, which contributes to our understanding of consumer waste behavior and consumer perception of food waste behavior, was dominated by qualitative research. However, the findings are based on a small sample and are not statistically representative [65,66]. Researcher empirically tested the influence of drivers and interventions of food waste reduction. Although recent studies attempt to extend the existing literature through a quantitative research approach to study the drivers of the behavior to reduce food waste [2,15,16], literature regarding sensitization of consumers and promoting waste reduction behaviors among consumers still reveals potential limits, specifically on household waste [12,14,67].

#### 1.3.3. Contextual Gap

Contextually, this study seeks to address the above-mentioned gap examining the determinants of household food waste reduction behavior of young Pakistani consumers. Prevention of food wastage depends upon the consumption practices of the customer. Consumer food wastage practices are understudied in the context of developing countries such as Pakistan. Furthermore, this research focuses exclusively on young Pakistani consumers. A large number of Pakistanis face malnutrition and food insecurity, where immediate action and consideration are needed to reduce the effects of food waste [68]. It has been found that around 1.3 billion tons (approximately one-third of the food for human consumption) are wasted in Pakistan [21]. A report by the World Wide Fund for Nature (WWF) confirms that 250 million tons of garbage in Pakistan is in the form of food scraps, pet bottles, and plastic bags, out of which 65% ends up on the coast [69]. The major contributor of solid waste in Pakistan is food waste at around 30% [70]. Research corroborates that around 36 million tons, which is about 33% of food, is wasted in Pakistan [22]. Media citing authorities reported that 60% of the population is food insecure and 44% of children under the age of 5 years are stunted [13]. Moreover, demographically, young consumers (18 to 33 years of age) represent a huge segment of the consumer market in Pakistan at around 51% [71]. Thus, findings from Pakistan can provide an improved worldwide view of the existing literature, since most of has been produced in western countries. It was also noted that young consumers as a target group of study may provide some unique results [72].

Existing research reveals contradictory results regarding the wastage behavior of young consumers. Some scholars claim that young consumers have more tendencies toward food waste compared to older generations [73]. This claim can be explained since older people are more educated on food management along with the experience of food storage situations, for example, during World War II [62]. It was also found that the lifestyle of the younger generation’s habit of eating out may have some serious effect on food waste [74]. On the contrary, recent research proved that younger consumers are far more concerned with food waste, particularly in developing countries [43], and understand the importance of recycling [44]. 

Young people engage in a wide range of food waste behaviors, from conspicuous food waste [75,76] to zero-waste movement engagement and advocacy [77,78]. It is necessary to conduct in-depth research to develop practical recommendations for policymakers because this group is highly heterogeneous [79].

## 2. The Theoretical Background

This research is unique in that it integrated two theoretical lenses theory of interpersonal behavior (TIB) and a comprehensive model of environmental psychology to explain emotional and cognitive factors associated with the food waste reduction behavior in a household context. The validity and efficiency of the theoretical lens, such as the theory of planned behavior, are questionable. The theory of planned behavior may not be sufficient to explain the complexity of behavioral intentions related to food waste. This research theorized that the theoretical lens of TIB and a comprehensive model of environmental psychology in one comprehensive theoretical framework could best explain the food waste reduction behaviors.

Research has identified multiple drivers and motivating factors of food waste reduction behavior and a demand for the development of a comprehensive model to address the complexity of such behaviors [14,16]. The theory of planned behavior (TPB) is frequently used to study consumer behavior in a food waste-related context [52,67,80]. TPB is based on a cognitive approach to predicting human behavior [81]. Given the plurality of food waste research, however, the explanatory powers of TPB with food waste are called into question by observers [14,50,80]. TPB ignores the emotional aspects of food waste that have been identified as significant in recent literature [47,52]. When including the emotional aspects by integrating different theoretical approaches, such as environmental psychology, interpersonal behavior theory, the theory of social practice, we can fully predict consumer behavior.

This new study expands on existing knowledge about young consumer waste reduction at the household level by identifying areas warranting further investigation. In this study, food waste reduction behavior is measured by using the reflections of the TIB and environmental psychology. TIB offers a theoretical lens to integrate cognitive, social, and emotional aspects [82]. This study attempted to integrate TIB and environmental psychology perspectives to examine emotional aspects (anticipated guilt), social aspects (sense of community), perceived consequences (awareness about consequences), external barriers (perceived busyness), and 3Rs. Prior literature has examined the significant role of food waste drivers such as anticipated guiltiness [83,84,85], awareness of consequences [86], sense of community [87,88] and environmental knowledge [47]. In the current study, there are four determinants of waste reduction behaviors. Anticipated guilt is an inner inhibitor that motivates individuals to consider their wasteful behaviors and find compliance with the standards and abstract norms of society [85]. Sense of community represents the social perspective of socially responsible actions [89]. Sense of community elicited a deeper sense of membership and encouraged individuals to act in a socially responsible manner and consider the consequences of their actions for future generations.

Researchers acknowledge structural constraints, such as the time that inhibits responsible behavior in different contexts [90]. Individuals evaluate benefits gained and sacrificed to engage in an activity. Applied in the context of food waste, consumers evaluate time availability (perceived busyness) and perceived cost in terms of monetary resources to act in a favorable manner [56]. Awareness about consequences is an important aspect of reducing food waste [91]. Individuals having awareness about consequences are more inclined towards moral obligations and act favorably. Awareness about consequences present contradictory results and require validation for the generalizability of results in a food context [92].

We have some practical significance in our study. For instance, food waste reduction practices can provide practitioners and policymakers with the opportunity to develop and implement customer education programs to minimize the adverse effects of food waste. This study contributes to policymakers’ agendas, as the Sustainable Development Goals (SDG) 2030 plan of action, which includes food security and improved nutrition [92]. The Ministry of National Food Security and Research Government of Pakistan has come up with the vision ‘A Food Secure Pakistan’. Furthermore, the Pakistan planning commission has also placed this challenge in vision 2025 and set a target to reduce the food insecure population from 60% to 30% [93]. It has been noted that the use of emotions may enhance the message persuasion and is more helpful to change the behavior [94]. Furthermore, this study examines the behavioral intentions of young consumers which have previously produced contradictory results. Research scholars and practitioners are more interested in monitoring the attitude of young consumers toward food waste for developing educational programs to limit food wastage [95]. 

The complex nature of human behavior is very difficult to predict [49]. Researchers found some reasons and behaviors that precede food waste and call for the formation of a theoretical framework to address the complexity of such behavior [16]. For instance, the TPB is a widely used theory for analyzing behavioral intentions regarding food waste context [67,80]. However, recent studies found some limitations regarding the explanatory power of TPB. TPB adopts a cognitive approach to explore human behaviors [81] whereas more research has been called to explain the emotional elaboration of customers regarding food wastage [47,52]. TIB takes into account emotions along with cognitive and social aspects to predict behavioral intentions in a comprehensive manner [96]. Richard et al. [97] established in their research that within the limits of Ajzen’s TPB framework, anticipated affecting independently forecasted behavioral intentions regardless of attitudes (evaluations) behind that behavior. Further, they differentiate clearly between effect and evaluation, thus limit the use of the term ‘attitude’ for overall evaluation response. 

**Figure 1:**
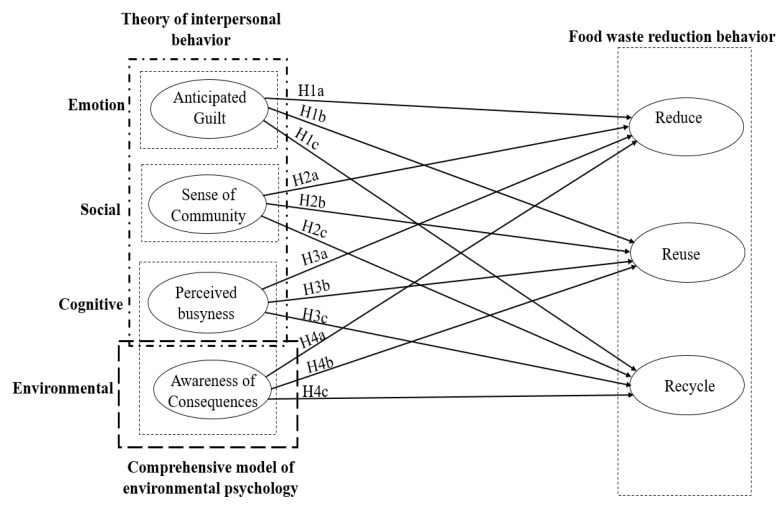
Conceptual Model of Food Waste Reduction Behavior.

The dire need to measure the features of behaviors regardless of their valence, causes, and differentiation is suggested by Lau-Gesk and Meyers-Levy [98]. Another aspect of theoretical contribution is the integration of TIB and environmental psychology. Although various literature from environmental psychology provides bases to model, key emotional and cognitive drivers comprehensively explain food-related behaviors. The conceptual model is presented in Figure 1.

## 3. Hypotheses and Conceptual Model

### 3.1. Hypotheses Development Regarding Consumer’s Waste Reduction Behaviors

In recent years, food waste has been discovered as one of the biggest issues and different researchers have developed different definitions and interpretations of the concept depending upon the scope, and objectivity of the study [99]. The term food waste is interchangeably used as food wastage and food loss [47]. Stefan et al. [100] considered all consumable products (eatables and drinkables) that have been discarded as food waste. Studies that are primarily focusing upon controlling or reducing the volume and quantity of food waste are termed quantity-oriented [101]. Conversely, studies where the nutritional value of food waste or sensory quality is focused, are termed quality-oriented studies [39]. Researchers also investigated the actual food loss considering quantity as well as quality [102] along with focusing its impact on society and environment [103,104,105]. Roodhuyzen, Luning [99] said that studies regarding food waste have also been differentiated based on voluntary and non-voluntary wastage, the extent to which food waste is conceived, and wastage in the early or later stage of the supply chain. Slorach et al. [33] offered different scenarios that can split food waste by 2030, but they also acknowledged that food waste reduction behaviors stand as the most effective way of sustainable food waste management.

Food wasted during consumption at the household level is to be considered as the most contributing factor and requires a complex set of management behaviors [14]. Hence, in order to facilitate practitioners and managers, this research emphases developing a comprehensive model to address the behavioral perspective of consumer food waste reduction behavior. Furthermore, food waste reduction behavior is operationalized as 3R (reduce, reuse, recycle) which is preferable to the traditional way of measuring food wasting intentions [59]. The factor of Reduce involves reducing waste by minimizing food disposal through careful planning and only buying food items required with well-organized eating and cooking practices [65]. It also involves the reusing of leftovers and food sharing [106]. While recycling involves the practice of recycling food waste through source separation and composting [107]. The increased importance of reducing food wastage in recent times has been directing researchers to consider recycling intentions [108,109]. In waste management, a number of studies adopted the concept of 3Rs to address waste behaviors such as plastic waste (e.g., [49], and food waste (e.g., [110]. Engagement of consumers in such behavior would be enhanced sustainably through elimination of or reduction in overall food waste [110].

#### 3.1.1. Anticipated Guilt

Anticipated guilt is recognized as the fundamental negative emotion for the development of affective-cognitive-action patterns of social norms to mitigate the caused damage [85]. The emotional facet, which independently contributes to the prediction of some intention is termed as an anticipated effect, where after performing a behavior, one develops positive feelings or negative emotions is known as the anticipated effect [111]. In their meta-analysis, Rivis, Sheeran [111] found that the association of anticipated regret with intentions is stronger than its association with generally anticipated emotion where regret is considered as experiential state and not one’s characteristics [112] which may bring negative consequences for longer periods as a trait of delayed cost effect. Researchers stated that regret or guilt will arise when considering the outcome of something [113] also elaborated on guilt as a reaction to some poor results and as director of behavior and strong motivator.

Kaiser and Differences [114] found its unique and significant contribution in determining intentions to act conservatively. Abraham and Sheeran [115] also proved it as a direct influencer for prospective behavior like exercising. Results of the meta-analysis by Sandberg and Conner [116] reflected that even attitudes were explained by levels of anticipated guilt. Therefore, Kim, Njite [117] rightly concluded that there is enough support empirically as well as conceptually that anticipated guilt could be assumed as a predictor of behavioral intentions. It was also found that anticipated guilt motivates young consumers to practices sustainable behaviors such as green banking [118], environmental sustainability, and food waste reduction behavior [46,85]. Hence, the following hypotheses are proposed considering the above-mentioned grounds:

**Hypothesis** **1a (H1a).** *Consumers with a higher level of anticipated guilt are more likely to reduce food waste.*

**Hypothesis** **1b** **(H1b).** *Consumers with a higher level of anticipated guilt are more likely to reuse the food left over.*

**Hypothesis** **1c** **(H1c).** *Consumers with a higher level of anticipated guilt are more likely to recycle food waste.*

#### 3.1.2. Sense of Community

Tajfel, Turner [119] explained that individuals wanted to be identified with their groups and consider themselves in group terms in the context of the social identity approach to collective action. The pride felt being a group member, symbols, values, and the fate shared either explicitly or implicitly is known as group identification [120]. Individuals perceive themselves as more depersonalized (i.e., not considering themselves unique anymore) when their social identity is ranked higher. Being identified with their group makes individuals feel, think and behave in accordance with their group norms and act according to group goals by bringing their self-perception and behaviors in line accordingly [121]. There is huge empirical support in the literature that collective action participation is strongly predicted by identification with groups [88]. The majority of people showed common interest and a high level of identification with the group instead of working for one’s goals and aspirations [120]. The organization has been demonstrated to be an effective motivator of participation by studies on collective action identification with the movement [122]. Despite the fact that food savers come from a variety of backgrounds and social classes, they have strong bonds and connections with one another, especially because of their common focus on the food issue (waste) but also because of their common goals (e.g., food waste reduction) and their moral standards. Research studies found that young consumers are more active in gaining a sense of community [59] and are more adoptive towards community behaviors [123] such as food waste reduction. Hence, the following hypotheses are proposed considering the above-mentioned grounds:

**Hypothesis** **2a** **(H2a).** *A higher level of sense of community drives customers to reduce food waste.*

**Hypothesis** **2b** **(H2b).** *A higher level of sense of community drives customers to reuse the food leftover.*

**Hypothesis** **2c** **(H2c).** *A higher level of sense of community drives customers to recycle food waste.*

#### 3.1.3. Perceived Busyness

Perceived busyness represents the prospect of an individual about the availability of time for certain actions [56]. It was found that busyness is a significant contributing factor in socially responsible behaviors [90]. Individuals are more inclined towards pro-environmental behaviors if they find more time available [124]. Time is an important factor to encourage environmental-friendly behaviors such as pro-environmental behaviors [125], recycling [90], and disposal of unused medicine [56]. In the context of household waste, it was observed that time constraints reduce the intentions of perfume waste separation [126]. Waste reduction behaviors require extra efforts and time to reconsider their wasteful behaviors and use food leftovers in a proper manner. Hence, the following hypotheses are proposed considering the above-mentioned grounds:

**Hypothesis** **3a** **(H3a).** *Perceived busyness restricts customers to reduce food waste.*

**Hypothesis** **3b** **(H3b).** *Perceived busyness restricts customers to reuse the food leftover.*

**Hypothesis** **3c** **(H3c).** *Perceived busyness restricts customers to recycle food waste.*

#### 3.1.4. Awareness about Consequences 

The altruistic model for studying behaviors assumes that one must be aware of its consequences as well [127]. Behaviors exhibited and their consequent outcomes are important to be considered while investigating consumer intentions. Individuals tend to develop and repeat positive attitudes towards those behaviors which may bring positive outcomes or consequences. This study explored consequences related to the environment that may emerge when waste products may be returned by individuals. Few researchers found that an awareness of consequences has a positive impact on return intentions [128], while some were able to establish an indirect effect on recycling intentions [129] and even a negative relationship with intentions was found by Tonglet, Phillips [130]. These contradictory results provide a basis for considering awareness of consequences in return intention research. It was found that awareness about the consequences of young consumers is significantly associated with food waste reduction behaviors [40]. Hence, the following hypotheses are proposed considering the above-mentioned grounds:

**Hypothesis** **4a** **(H4a).** *Consumers having more awareness about the consequences of food waste are more likely to reduce food waste.*

**Hypothesis** **4b** **(H4b).** *Consumers having more awareness about the consequences of food waste are more likely to reuse the food leftover.*

**Hypothesis** **4c** **(H4c).** *Consumers having more awareness about the consequences of food waste are more likely to recycle food waste.*

Now, we present in Figure 1 the model we used in our paper and include all hypotheses in the figure. Hence, the following hypotheses are proposed considering the above-mentioned grounds:

In the context of food waste, the current study analyzes waste reduction behaviors through four independent variables from emotional and cognitive perspectives. Hence, anticipated guilt, an emotion, becomes more relevant for the prediction of behavioral intentions [131]. Individuals being part of a social group seek compliance with the group they feel a sense of membership, and follow the standard of that group [132]. Perceived consequences may also motivate individuals to avoid wasteful behaviors [133]. As the availability of time or busyness increases, so does the likelihood of acting in a more responsible manner [56]. This research responds to these gaps and develops a comprehensive model to explain cognitive as well emotional responses toward food waste reduction in the context of household customers of Pakistan.

### 3.2. Measure and Methodology

#### 3.2.1. Measures

A two-section survey was developed based on well-established measurement scales. The first section was composed of items used to measure the constructs of the theoretical model. Each structure consisted of several items and participants were inquired to record their replies at a 5-point Likert scale ranging from 1 for strongly disagree to 5 for strongly agree. Anticipated Guiltiness was measured with 3 items scale adopted from Soorani and Ahmadvand [85]. Awareness about consequences was measured with a 5 item scale adopted from Khan, Ahmed [134]. Sense of community was measured with a 6 item scale adopted from Dixon, Deline [135]. Perceived busyness was measured with 4-time scales adopted from Foon et al. [56]. Intentions to reduce were measured with a 3 item scale adopted from Heidari, Mirzaii [14]. Intention to reuse was measured with 5 items scale adopted from Khan, Ahmed [134]. Intentions to recycle were measured with 5 items adopted from Khan, Ahmed [134]. Respondents were asked to rate their agreement on a five-point Likert scale (i.e., 1 = strongly disagree & 5 = strongly agree). The second section was designed to get information on the sociodemographic characteristics of respondents. A pilot test of the questionnaire was also performed to ensure that the content and face validity criteria were met.

#### 3.2.2. Methodology (Data Collection Procedure)

The full-scaled administrative survey data was collected from young Pakistani consumers of household food products since the high amount of food wastage in this country provides a study margin to give a more worldwide view in the existing literature. Household food products include vegetables, fruits, meat, poultry, seafood, pulses, and dairy products [68].

The young consumers are selected for this study based on several reasons: First, the demographically young consumer represents a huge segment at 51.25% of the consumer market in Pakistan [71]. Second, the young age group, being the future consumer, has the capability of making a difference in the coming decades [117]. Third, young consumers with a higher education may also have basic knowledge of the concept of food waste and provide more factual responses rather than hypothetical [79]. Therefore, it is significant to examine the viewpoint in which young Pakistanis behave towards food wastage.

Between November 2019 and February 2020, a public survey was conducted on a “next-to-last” basis using a purposive sampling strategy. The survey was conducted in public places in Pakistan’s major cities, with respondents being approached and asked to fill out a self-administered questionnaire voluntarily. Khan, Ahmed [49] and Islam, Attiq [136] found that this technique to be effective in attracting true respondents as well as avoiding non-serious respondents, thus offering a higher level of certainty in the results. This practice turned out to be extremely useful, as the overall response rate was 69.3% for a sample size of a total of 391 respondents.

## 4. Empirical Results

The demographic profile is presented in Table 1. From the table, we find that a total of 391 responses were received, out of which there were 44% females, 161 respondents were aged from 23 to 27 years, and the majority of respondents were highly educated people (Masters 131 (33.5%), and above Masters 153 (39.2%)).

Data were tested for missing values, outliers, and normality before conducting structural equation modeling. There were no missing values and outliers in the data set. All the items presented Skewness and Kurtosis within the expected range of ±3. Common method bias was tested by using Harman’s single-factor. The largest factor accounted for 40.012% less than the threshold value of 50%, an indication of no common method biases [137,138,139]. The results of collinearity test are presented in Table 2.

Furthermore, Kock and Lynn [140] and Kock and Gaskins [141] defined the full collinearity test as a comprehensive technique in which simultaneous assessment for both vertical and lateral collinearity is executed. The present study has generated the variance inflation factors (VIFs) for all latent variables in a model. Kock and Gaskins [141] indicated that if the VIF values are greater than 3.3, then the model is contaminated by common method bias. In the present study, common method variance was not observed as a significant threat as after observing the values from the table it is indicated that all the VIF values are less than 3.3, representing that the model is free from common method bias.

The partial least square structural equation modeling (PLS-SEM) technique was used to analyze the data. Hair, Sarstedt [142] recommended the procedure of evaluating outer and inner measurement followed by hypothesis testing. The outer model involves the establishment of validity (i.e., convergent validity and discriminant validity) and reliability (i.e., composite reliability).

### 4.1. Measurement Model

Results of the outer model that are outer loads, Cronbach’s alpha, composite reliability (CR), and average variance explained (AVE) are shown in Table 3. Consistent with the recommendation of Hair, Sarstedt [142] results are in support of reliability, convergent validity, and discriminant validity. Results are presented in Table 3. For the statement of each item, see Appendix A (Table A1).

Discriminant validity indicates that variables that should be unrelated are unrelated. In other words, discriminant validity is used to study the difference among latent variables. Fornell and Larcker [143] criterion is used to test the discriminant validity. This criterion matches the square root of the AVE of all variables with correlation values among the latent variables. Results are presented in Table 4 showing that the square root of the AVE of each latent variable is higher than their respective correlation values [144]. Furthermore, this table contains the mean values of study variables. The mean value of variables (i.e., anticipated guilt, sense of community, awareness of consequence, reduce, reuse, and recycle) is above 3, which shows the respondents have a tendency toward agreement on the Likert scale. While the mean value of perceived busyness is 2.04, this shows the respondents are on the ‘disagree’ side according to the 5-point Likert scale.

### 4.2. Structural Model

Satisfactory results of the outer model are an indication to proceed towards inner model evaluation. The inner model is estimated by employing bootstrapping. The quality of the inner model is dependent upon the capability to predict the endogenous construct [145]. The quality of the inner model is assessed by using the criterion of collinearity analysis, coefficient of determination R2 analysis, and predictive relevance analysis (Q2) which are carried out [146]. To test the collinearity variance inflation factor (VIF), it was estimated, and the values of VIF are below the cut-off point of 3.3. This represents that collinearity does not prevail [147]. R2 measures how close the data are to the fitted regression line. The value of R2 lies between 0 and 1. The higher value shows a good model fit for data. R2 values are 0.47, 0.56, and 0.57 for reduce, reuse, and recycle, respectively. Q2 quantifies the predictive relevance of the model. The values of Q2 are 0.30, 0.31, and 0.35 for reduce, reuse, and recycle, respectively. These values are higher than the threshold (i.e., >zero) [144]. Results are presented in Table 5.

**Figure 2:**
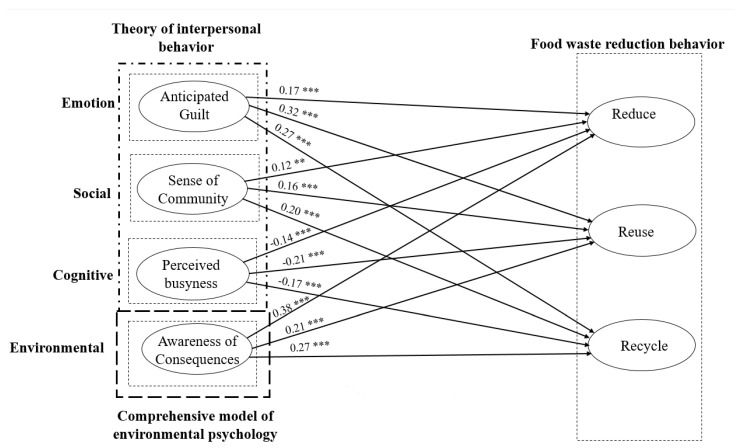
Structural Model. Notes: *** *p* < 0.001, ** *p* < 0.01.

Structural path analysis is performed to test the hypotheses. The results of the hypotheses testing are presented in Table 6 and Figure 2. The results of path coefficients indicated that anticipated guiltiness has a significant impact on reduce (β = 0.17, *p* < 0.00), reuse (β = 0.32, *p* < 0.00), and recycle (β = 0.27, *p* < 0.00), supporting H1a, H1b, and H1c, respectively. The findings revealed anticipated emotions that are regret as an important contributing factor in behavioral formation. It can be observed that more negative emotions trigger more positive behavioral responses in societal as well as environmental aspects. The feeling of guilt forces them to act in an ethically and socially responsible manner and motivates them to reduce food wastage. In line with the altruistic behavior model, awareness of consequences was found as a contributing factor towards food waste reduction behavior [128].

Results in Figure 2 depict that when consumers are aware of the consequences of food waste reduction, they are more participative towards food waste reduction. The reorganization of consequences such as economic burden, needless hunger, and climate change induce customers to act in a sustainable manner and contribute towards food reduction practices. The greater involvement of consumers in planning for reducing consumption, reusing consumption, and recycling of leftovers mitigate the hazardous effects of food waste. A significant and positive impact of the sense of community was found on reduce (β = 0.12, *p* < 0.04), reuse (β = 0.16, *p* < 0.00), and recycle (β = 0.21, *p* < 0.00), supporting H2a, H2b, and H2c, respectively. The results of the study are also in support of the significant impact of a sense of community towards waste reduction behavior. The findings are in line with the study of Dixon et al. [135] and Schanes and Stagl [88]. The feeling of affiliation with society stimulates customers to actively consider society and respect the opinion of others [80]. Thus, customers with a higher level of sense of community activities are more able to avoid the socially harmful effects of food waste. 

Furthermore, the result represents that perceived busyness has a negative effect on reducing (β = −0.14, *p* < 0.00), reuse (β = −0.21, *p* < 0.00) and recycle (β = −0.17, *p* < 0.001) in support of H3a, H3b, and H3c, respectively. It was also found that consumers with higher levels of perceived busyness significantly contribute to food waste reduction behavior. The finding of the study validates the results of the existing research [56,125]. It represents that availability of less time and being busy restricts individuals from being involved in food waste reduction behavior. A lack of time served as an obstacle to favorable behavioral intentions. Results regarding awareness of consequences showed a significant and positive impact on reduce (β = 0.38, *p* < 0.00), reuse (β = 0.21, *p* < 0.00), and recycle (β = 0.27, *p* < 0.00), in favor of H4a, H4b, and H4c, respectively.

## 5. Discussion and Concluding Remarks

In this paper, we conduct an in-depth study on food waste reduction behavior by using the 3R model to conduct an empirical analysis to understand antecedents of food waste reduction behaviors, including Reduce, Reuse, and Recycle. More specifically, this study has examined the impacts of emotional (anticipated guilt), social (sense of community), cognitive (perceived busyness), and environmental factors (awareness of consequences) on food waste reduction behavior. We investigate the final tier of the food waste supply chain, which is the customers, since the impact on society and the environment of household food waste at consumers’ level is one of the most important issues. This study examines the main conceptual and methodological gaps and reveals the behavior of young household consumers regarding food waste reduction.

To do so, we hypothesize that consumers who emotionally feel guilty for wasting food are more likely to engage in food reduction behaviors such as Reduce, Reuse, and Recycle. The second hypothesis was related to a person’s social identity with groups working for the reduction of food waste behaviors. It was hypothesized that a higher sense of community will be positively related to food waste reduction behaviors. Our findings are in line with notions of both anticipated affect and moral norms’ impact on how people intend to behave [111]. More specifically, when people anticipate certain emotions, they tend to mold their intentions and behaviors. Secondly, moral norms signal to them through social learning to alter their behaviors. This study, thus, concludes that when consumers have anticipated guilt about wasting food and feel pressure from the community not to engage in food waste, it has an ultimate impact on food waste reduction behaviors.

The third hypothesis set in our paper considers time constraints as an important predictor of food waste reduction behaviors, such that people who perceive themselves as busier may not be inclined to engage in food waste reduction behaviors. More specifically, a negative relationship has been hypothesized between perceived busyness and food waste reduction behaviors. Time constraints are an important determinant of consumer perceptions and interpretation [148]. When people perceive themselves as busy and have a lack of spare time, they ignore very important convictions [149].

The fourth hypothesis set in our paper considers environmental consciousness as an antecedent to food waste reduction behavior. We hypothesize that awareness about environmental consequences will have a positive impact on food waste reduction behavior. Our finding supports the notion that consumers’ awareness about consequences fosters value orientations which, in turn, develop behaviors that cognizant of socially accepted norms [150].

This study integrates the theories of both interpersonal behaviors [82] and environmental psychology perspectives to examine consumer’s emotional, social, perceived consequences, external barriers, and the 3Rs. Although the theory of planned behavior (TPB) is a popular theory to predict consumers’ intentions and behaviors, TPB has been previously criticized for its failure to incorporate emotional and environmental factors in the context of food waste reduction. To circumvent its limitation, this study has taken into consideration relevant emotional, social, environmental, and personal factors to comprehensively explain and predict consumer food waste behaviors. From methodological perspectives, this study has applied quantitative techniques and recruited young consumers as respondents in this study. Data collected through questionnaires have been analyzed through structural equation modeling by using Smart PLS software. The analysis has further strengthened the methodological validity and rigor of this study.

Our findings infer that when consumers are aware of the consequences of food waste reduction, they are more participative towards food waste reduction. Our findings are consistent with the reorganization of effects such as economic burden, needless hunger, and climate change, which induce customers to act sustainably and contribute towards food reduction practices [150]. Our findings also support the argument that the greater involvement of the consumers in planning for reducing consumption, reusing consumption, and recycling of leftovers mitigate the hazardous effects of food waste. The feeling of affiliation with society stimulates customers to actively consider society and respect the opinion of others [80]. Thus, customers with higher levels of sense of community activities, in a more favorable manner, avoid the harmful social effect of food waste [87,88]. Our findings also support the proposition that waste reduction behaviors require extra efforts and time to reconsider their wasteful behaviors and properly use food leftovers. Recent research from Malaysia, for example, also found that reducing food waste has a greater role in sustainable food waste management [151].

Our findings obtain some important insights about consumers’ food waste reduction behaviors. For example, we find that food waste reduction behavior is a multi-pronged phenomenon in which individuals’ emotional state, social appraisal, cognitive perceptions, and environmental consciousness play an important role in developing behavioral intentions towards sustainable food waste reduction. This finding supports the arguments from both [14,15]. More specifically, according to Stefan, van Herpen [100], food reduction positioned in the minds of individuals as a must-do job may increase a guilty feeling in case of overuse of food. This guilty feeling will activate some mental processes which, in turn, will encourage individuals to participate in food reduction behaviors. 

Secondly, if food reduction behavior is promoted socially, it will develop a community of practice that will further motivate individuals to comply with social norms and a sense of community that engages individuals in food reduction behaviors [152]. However, in this situation, individuals’ understanding of time constraints and hurrying nature may be threatening in the sense that it could have a negative impact on food reduction behaviors [149]. Finally, environmental awareness plays an important role in encouraging food reduction behaviors [150].

This study also has implications for academics, practitioners, and policymakers. For academics, this study could help them understand that food reduction behavior is a multi-pronged issue and is well-rooted in different factors. Therefore, efforts to develop a comprehensive model covering all relevant aspects are necessary. This study has made a good effort in this regard. For example, the integration of TIB and environmental psychology resulted in the theoretical advancement of examining consumer behavior intentions in the context of household food wastage. The results of the study are based on the responses of young consumers who are the best representatives and relevant samples for the investigation of household sustainable food waste reduction behavior. Young consumers being the future may serve as a proxy of the global population. It was found that the concept of 3Rs (i.e., reduce, reuse, and recycle) is helpful in addressing the complexity of food reduction behavior. Another theoretical contribution of our study is the inclusion of emotional and external aspects to overcome the predictive limitation of TPB in this context. 

For practitioners, it is necessary to encourage young consumers to participate in food reduction behaviors by increasing the importance of food safety and inculcating in the minds of young consumers about the hazards of food waste. Practitioners must develop methods to curtail food waste by devising promotional messages keeping in view different aspects identified in this study. For both practitioners and policymakers, this study has many implications. First, the results of the study show that guiltiness in education programs and publicity strategies needs to be included. Specifically, with socially prescribed behaviors that include sustainable food waste, the inclusion of guiltiness becomes more effective. Recognizing regrets arising from unnecessary, natural, and economic resources-inherent food waste can develop a deeper sense of error and jeopardize others by individual behavior.

In addition, policymakers will benefit from this study to raise the awareness of young consumers about food reduction. Policies may be formulated after identifying key areas where most of the food is wasted unnoticed. Young consumers may be aware to engage in sustainable food waste reduction behaviors by following policies and procedures developed in these regards. Thus, programs and advertisements that focus on societal responsibility would have a better chance of motivating consumers to do their part in helping prevent food waste in the area of sustainability that would also have to anticipate feelings of guilt in advance. Consumers should learn about the positive effects of eliminating food waste in order to transform behavior towards food waste. Knowledge of the economic losses, damage to the environment and social problems caused by food waste motivates consumers to prevent food waste. In addition, it encourages community members to develop a sense of association and to contribute to the sustainable reduction of food waste. The exhibit of extreme environmental degradation is also very helpful in designing better customer appeal to transform behaviors to reduce sustainable food waste. Although, this paper has collected data from young consumers of Pakistan who enjoy a greater share of the population profile of Pakistan. Data were collected through a questionnaire method and we analyzed the data by using the structural equation model. This serves the purpose of methodological rigor. Nevertheless, this study has some limitations as well. Firstly, a cross-sectional study collecting data in a one-shot cannot explain causality for a long period. Secondly, the sample size used in this paper may not be a good representative of the population. Thirdly, respondents were mainly highly educated people who may have sugar-coated responses to give more favorable responses to the study questions due to the realization of the importance of the topic. In this regard, people who are involved in household food waste may be housewives and maids. Responses from such a relevant sample may provide a new avenue of direction. Another limitation is that this study has used the theory of planned behavior although the inclusion of emotional and environmental factors as responses to criticism levied against TPB could be a strength of this study.

Moreover, some other theories could also be used in predicting sustainable food waste behaviors well. In addition, this study takes into consideration only emotional aspects, cognitive, social, and environmental aspects. All these aspects reflect one’s understanding, emotions, thinking, perception, and level of awareness. However, actual practices such as cooking, storing, packaging, and eating behaviors are ignored in this study. Future research could incorporate these factors into the study. Moreover, future studies could be conducted by using experience designs collecting data from some relevant respondents who are mainly engaged in cooking, serving, ordering, and eating food. Other factors such as a show of status, richness, generosity, and others that allow people to display broader behaviors about ordering and serving food to others could also be causes of food waste. This is also a good future direction of the study.

This study focuses on the factors that may affect waste reduction behavior by using the self-reported measures that may lead to some biases. Respondents may exaggerate their intentions towards sustainable food waste, which may have an impact on the quality and generalizability of results [153]. Future research could consider actual waste reduction behavior to increase the generalizability by exploring the link between intentions and behavior. This study only examined the effect of factors that may precede waste reduction behavior and did not explore the ways for reducing food waste that may facilitate more to address the problem. Thus, future research may adopt various qualitative methods to find out the ways and practices for waste prevention. It is also suggested that some other variables like financial attitudes, religion, and food expenditures could also be used to increase the explanatory power of the model. Furthermore, sociodemographic characteristics like psychographics, size of household, family life cycle, and income may also provide some important findings. The results of this study were based on young consumers, some research found differences among the behaviors of the young population itself. The young people in the age group of 18 to 24 are considered to be less educated towards sustainable food waste and storage, and usually less concerned about the consequences of sustainable food waste. At the same time, young adults may have greater knowledge about sustainable food waste consequences and the monetary value of food waste so that they are more inclined towards food reduction behavior. Thus, future research could explore the difference in food reduction behavior among different age groups.

## Figures and Tables

**Table 1:** Demographic Variable Profile.

Demographic	Category	Frequency
Gender	Male	219 (56%)
	Female	172 (44%)
Age (In years)	18–22	119 (30.4%)
	23–27	161 (41.2%)
	28–32	88 (22.5%)
	33 and above	23 (5.9%)
Education	Matric	11 (2.8%)
	Intermediate	26 (6.6%)
	Bachelor	70 (17.9%)
	Masters	131 (33.5%)
	Above Master	153 (39.2%)
Monthly Income	Below 15,000	118 (30.2%)
	15,000–35,000	140 (35.8%)
	35,001–55,000	67 (17.1%)
	55,001–75,000	43 (11.0%)
	Above 75,000	23 (5.9%)

**Table 2:** Common Method Bias (Collinearity Test).

Variables	ANG	SOC	PBY	AOC	RED	REU	REC
ANG		2.17	2.17	1.98	2.11	2.06	2.13
SOC	1.91		2.40	1.89	1.92	1.91	1.87
PBY	2.14	1.82		2.05	2.14	2.09	2.13
AOC	2.26	2.47	1.61		2.19	2.44	2.41
RED	1.85	1.87	1.87	1.71		1.89	1.87
REU	2.62	2.77	2.71	2.71	2.72		2.26
REC	2.81	2.77	2.83	2.78	2.82	2.34	

Note: ANG = Anticipated guilt, SOC = Sense of Community, PBY = Perceived busyness. AOC = Awareness of consequences, RED = Reduce, REU= Reuse and REC = Recycle.

**Table 3:** Results of Measurement Model.

Measures	Reference	Code of Each Item	Outer Loading	Cronbach Alpha	CR *	AVE *
Anticipated Guilt	[85]	ANG1	0.86	0.80	0.88	0.72
		ANG2	0.87			
		ANG3	0.81			
Sense of Community	[135]	SOC1	0.80	0.87	0.91	0.60
		SOC2	0.81			
		SOC3	0.79			
		SOC4	0.76			
		SOC5	0.75			
		SOC6	0.74			
Perceived Busyness	[56]	PBY1	0.79	0.77	0.85	0.59
		PBY2	0.82			
		PBY3	0.63			
		PBY4	0.82			
Awareness of Consequences	[134]	AOC1	0.77	0.84	0.89	0.61
		AOC2	0.80			
		AOC3	0.79			
		AOC4	0.81			
		AOC5	0.77			
Reduce	[14]	RED1	0.81	0.78	0.87	0.70
		RED2	0.88			
		RED3	0.81			
Reuse	[134]	REU1	0.75	0.84	0.88	0.61
		REU2	0.80			
		REU3	0.77			
		REU4	0.81			
		REU5	0.78			
Recycle	[134]	REC1	0.79	0.87	0.90	0.66
		REC2	0.81			
		REC3	0.83			
		REC4	0.84			
		REC5	0.81			

Note: CR = Composite reliability, AVE = Average variance extracted, * significant at 5%.

**Table 4:** Discriminant Validity and Correlation Analysis.

Constructs	Mean	SD	1	2	3	4	5	6	7
Anticipated Guilt	3.75	0.78	0.85	-	-	-	-	-	-
Sense of Community	3.95	0.68	0.46	0.77	-	-	-	-	-
Perceived Busyness	2.04	0.69	0.46	0.64	0.77	-	-	-	-
Awareness of Consequences	3.67	0.75	0.65	0.45	0.55	0.79	-	-	-
Reduce	3.66	0.83	0.54	0.47	0.51	0.63	0.84	-	-
Reuse	3.82	0.67	0.64	0.55	0.59	0.62	0.51	0.78	-
Recycle	3.80	0.72	0.62	0.57	0.57	0.64	0.56	0.74	0.81

**Table 5:** Results of Structural Model.

Constructs	VIF			R2	Q2
	Reduce	Reuse	Recycle		
Anticipated Guilt	1.84	1.84	1.84	-	-
Sense of Community	1.80	1.80	1.80	-	-
Perceived Busyness	1.99	1.99	1.99	-	-
Awareness of Consequences	2.03	2.03	2.03	-	-
Reduce	-	-	-	0.47	0.30
Reuse	-	-	-	0.56	0.31
Recycle	-	-	-	0.57	0.35

**Table 6:** Summary of all Hypotheses Result.

Hypothesis	Path	β	*p*
H1a	Anticipated Guilt→Reduce	0.17	<0.00
H1b	Anticipated Guilt→Reuse	0.32	<0.00
H1c	Anticipated Guilt→Recycle	0.27	<0.00
H2a	Sense of Community→Reduce	0.12	<0.04
H2b	Sense of Community→Reuse	0.16	<0.00
H2c	Sense of Community→Recycle	0.20	<0.00
H3a	Perceived Busyness→Reduce	−0.14	<0.00
H3b	Perceived Busyness→Reuse	−0.21	<0.00
H3c	Perceived Busyness→Recycle	−0.17	<0.00
H4a	Awareness of Consequences→Reduce	0.38	<0.00
H4b	Awareness of Consequences→Reuse	0.21	<0.00
H4c	Awareness of Consequences→Recycle	0.27	<0.00

## Data Availability

Data are contained within the article.

## Appendix A

**Table A1:** Items of each measure.

Measures	Items
Anticipated Guilt	ANG1: I feel guilty
	ANG2: I feel guilty due to food wasting while many people do not have assured access to edible food
	ANG3: I will properly manage and take care of my future behaviors to reduce feeling of guilt
Sense of Community	SOC1: I have roots in this place
	SOC2: My neighbors often help me if I need it
	SOC3: I feel like I am a member of the community
	SOC4: I feel I am a member of the people who work within university/college
	SOC5: I feel I am a member of the people who work within my department
	SOC6: I feel I am a member of the people work within work within my organization
Perceived Busyness	PBY1: I am a busy person
	PBY2: I have less time on my hands than the average person
	PBY3: I feel like I am rushing too often
	PBY4: I have very little free time
Awareness of Consequences	AOC1: In my opinion, food waste reduction is a major way to reduce pollution
	AOC2: I consider that food waste reduction creates a better environment for future generations
	AOC3: I believe that food waste reduction is a major way to reduce wasteful use of landfills
	AOC4: I consider that food waste reduction is a major way to conserve natural resources
	AOC5: I think food waste reduction saves money
Reduce	RED1: In the next few weeks, I plan to reduce my food waste with more attention to buying
	RED2: In the next few weeks, I plan to reduce my waste of food with more attention to my meals
	RED3: In the next few weeks, I plan to get more information about the effects of food waste on the environment and the economic and social conditions of my community and my community
Reuse	REU1: I reuse leftover food because it can significantly benefit the environment
	REU2: I reuse leftover food for other purposes to get the most out of them
	REU3: I reuse leftover food to save money
	REU4: I reuse leftover food rather than buying them new
	REU5: I try to reuse leftover food for other purposes because throwing away significantly contributes to the landfill problem
Recycle	REC1: I resell much of my leftover food for economic reasons
	REC2: I resell leftover food to recycle
	REC3: I resell leftover food to save money
	REC4: I resell leftover food to reduce landfill problems
	REC5: I resell my unwanted food rather than throw it away
